# 

**Published:** 1886-08

**Authors:** 


					﻿hales*
aloVRN/XL'J
HEALT h
ESTABLISHED 18^4
Vi- 33
Ui- 8
OFFICE: 206 BROADWAY, EVENING POST BUILDING, Boom 11. N. Y.
Entered as Second-class Matter at the Post-Office, New York.
				

